# Electroencephalography Network Effects of Corpus Callosotomy in Patients with Lennox–Gastaut Syndrome

**DOI:** 10.3389/fneur.2017.00456

**Published:** 2017-09-04

**Authors:** Jun-Ge Liang, Dongpyo Lee, Song Ee Youn, Heung Dong Kim, Nam-Young Kim

**Affiliations:** ^1^RFIC Center, Kwangwoon University, Seoul, South Korea; ^2^Epilepsy Research Institute, Yonsei University College of Medicine, Seoul, South Korea; ^3^Department of Pediatrics, Padiatric Epilepsy Clinic, Severance Children’s Hospital, Yonsei University College of Medicine, Seoul, South Korea

**Keywords:** Lennox–Gastaut syndrome, functional network effects, corpus callosotomy, small-world structure, electroencephalographic, functional connectivity

## Abstract

**Objectives:**

This study aimed to investigate the functional network effects of corpus callosotomy (CC), a well-recognized palliative surgical therapy for patients with Lennox–Gastaut syndrome (LGS). Specifically, we sought to gain insight into the effects of CC on LGS remission, based on brain networks in LGS by calculating network metrics and evaluating by network measures before and after surgery.

**Methods:**

Electroencephalographic recordings made during preoperative and 3-month postoperative states in 14 patients with LGS who had undergone successful CC were retrospectively analyzed. First, undirected correlation matrices were constituted for the mathematical expression of functional networks. Then, we plotted these networks to analyze the effects of CC on connectivity. In addition, conventional local and global network measures were applied to evaluate differences in network topology between preoperative and postoperative states.

**Results:**

In the preoperative state, hubs were mainly distributed around the paramedian regions. After CC, the hubs moved from the paramedian regions to the dual-hemisphere and even the lateral regions. Thus, the general connectivity state became more homogeneous, which was verified by network plots and statistical analysis of local measures. The results of global network measures indicated a decreased clustering coefficient in the delta band, decreased characteristic path length in both the delta and gamma bands, and increased global efficiency in the gamma band.

**Conclusion:**

Our results showed a consistent variation in the global brain network that converted to a small-world topology with an optimal balance of functional integration and segregation of the network. Such changes were positively correlated with satisfactory surgery results, which could be interpreted as being indicative of LGS recovery process after CC. For patients with refractory LGS along with no focal epileptogenic zone findings, which were not suitable for the resective surgical therapy, our results verified that CC could work as an effective surgical treatment option.

## Introduction

Lennox–Gastaut syndrome (LGS) is one of the most severe childhood-onset epilepsies (1, 2). Epilepsy in LGS is usually medically refractory and generalized with multiple types of seizures observed. Additional characteristics of LGS are atypical electroencephalographic (EEG) properties (e.g., generalized slow sharp, wave discharges, and generalized paroxysmal fast activities) and progressive mental retardation (3). Most patients with LGS continue to experience seizure throughout life despite therapy with anti-epileptic drugs (AEDs), and patients also manifest cognitive, psychiatric, and behavioral problems (4, 5).

Corpus callosotomy (CC) is considered as a palliative surgical treatment for LGS when treatment with AEDs fails and if there is no evident focal pathologic brain region treatable by resective surgery (6, 7). Accumulated evidence indicates that favorable outcomes can be achieved through CC in patients with LGS who suffer from medically intractable seizures with abundant generalized and multiregional EEG abnormalities (8, 9). Major benefits of CC appear to be twofold. First, because the corpus callosum is a critical pathway for the interhemispheric spread of epileptic activity, disconnection between cerebral hemispheres can modify seizures such that spread is slow, and thereby provide patients a warning that allows them to protect themselves. Second, in instances where seizure expression requires bilateral synchrony, disruption of this synchrony may potentially eliminate this seizure type (6, 10).

Recent studies investigating functional networks in patients with epilepsy tend to focus on elucidating the contributions of specific brain regions to integral network patterns (11–13). This research is rooted in the concept that the global brain can be regarded as a network formed by interconnections between all of the brain’s regions, a concept based on a large body of work in neuroscience (14–16). Researchers have increasingly studied brain network properties in the context of behavioral research, psychological studies, and the diagnosis and treatment of neurological and psychiatric diseases (17, 18). Compared to basic connectivity measures, which only provide information on how pairs of different brain regions are functionally connected, network analysis characterizes structural alterations in the global brain network, which provides valuable information on seizure onset, propagation, and termination in patients with LGS (19). Small-world network models are commonly used and highly efficient in achieving optimal characterization of local and distant connections (20). Enhanced signal propagation and synchronizability across small-world networks are usually regarded as a seizure termination pattern, while a more regular pattern characterizes the ictal state. This may have implications for LGS, as it is considered to be a cerebral network disease. For instance, it has been shown that there is a markedly different functional connectivity pattern between healthy subjects and patients with LGS. In addition, patients with epilepsy associated with cognitive decline show less efficient network patterns compared to healthy controls (11).

According to previous studies (21–23), most patients who have generalized seizures and drop attacks experience alleviation of morbidity and incidence of epilepsy through CC. Considering CC from the brain network perspective, disconnection of corpus callosum cuts off the main connective paths between the two hemispheres, which should have a significant impact on the topology of the entire network. Given that CC can successfully treat various seizures and epilepsy syndromes, there may be an association between the effects of brain network state and this operative scheme. However, the types of network effects caused by CC and their influence on clinical outcomes have not been well studied.

The aim of this study was to investigate the brain network effects of CC on LGS patients. Herein, we provide evidence forming the basis of a hypothesis that a favorable CC outcome may positively relate to a small-world brain network with simultaneously high segregation and integration, as well as improved inner-communication efficiency. In addition, we find that some consistent variations based on local and global network measures after CC, which might relate to the LGS remission process conveyed by CC.

## Materials and Methods

### Patient Selection and Evaluation

We enrolled 14 patients who underwent CC with favorable surgical results between 2009 and 2012 at Severance Children’s Hospital of Korea. The patients were selected according to the following inclusion criteria: (1) preoperative epileptiform discharges were typical of EEG finding of LGS: generalized slow sharp and waves and generalized paroxysmal fast activities with slow and unorganized background; (2) brain MRI findings without definite brain lesions; (3) not a candidate for focal resection based on clinical judgment and interictal EEG recording; (4) markedly improved seizure condition after surgery. We did not include the Lennox–Gastaut phenotype patients who showed some but not all of the electrical features of the LGS (2, 3, 24).

The study was approved by the institutional review board of Yonsei University, College of Medicine, Seoul, Korea and written informed consent was obtained from all parents of the children prior to participation. Table 1 summarized the clinical characteristics of the patients involved in this study. In short, patients showed improved EEG patterns such as reduced frequency of generalized epileptiform discharges and lateralization or localization of epileptiform discharges. The number of medications was reduced or remained same except for two patients. All but one patient showed reduced seizure frequency after CC and three of them achieved seizure free.

**Table 1:** Clinical characteristics of Lennox–Gastaut syndrome patients.

Patient no.	Gender/age	Seizure onset age	Main seizure type	Preoperative medication	Postoperative medication	Preoperative electroencephalographic (EEG)	Postoperative EEG	MRI	PET	SPECT	Outcome
1	F/8	2	GTC, head drop	LMT, ZNS, LEV, VPA	LMT, LEV, CBZ	GPFA, GSSW, MSWD	Rt LPFA, Rt F SWD	Mild brain atrophy	Rt FPT ▾	Rt FPT ▾	Seizure free
2	M/4	0.5	GTC, jerking	VGB, LEV	VGB, LEV	GPFA, GSSW	GPFA, GSSW ▾	Suspicious Rt F pachygyria	Rt F ▾	Rt F▾	90% decrease
3	F/15	1.6	GTC, jerking	TPM, OXC, VGB	TPM, OXC, VGB	GPFA, GSSW	Lt F SWD	Multiple non-specific Rt WMH	Non-specific	Non-specific	95% decrease
4	M/7	0.8	GT	ZNS, VPA, LEV	ZNS, VPA	GPFA, GSSW	Lt LPFA	Rt OT WMH	Rt P ▾	Non-specific	Seizure free
5	M/2	0.8	Spasms	VGB, LEV, VPA	LEV, TPM	GPFA, GSSW	Lt LPFA, Lt SWD	Diffuse brain atrophy	Normal	Rt F ▴	60% decrease
6	M/12	8	GTC, head drop	LMT, LEV, TPM	LMT, LEV, TPM	GPFA, GSSW	Rt LPFA	Normal	Multifocal ▾	Non-specific	Seizure free
7	M/5	0.5	GT, head drop	LEV, CBZ	LEV, ZNS	GPFA, GSSW	Lt LPFA	Normal	Non-specific	Non-specific	90% decrease
8	F/6	0.1	Spasms	LMT, TPM	LMT, TPM	GPFA, GSSW	Lt LPFA	Normal	Lt H ▾	Lt H ▾	30% decrease
9	F/1	0.3	Spasms	TPM, VPA	VPA, TPM, ZNS	GPFA, GSSW	GPFA, GSSW ▾	Normal	Rt T ▾	Rt T ▾	50% decrease
10	M/10	6	Atypical absence, head drop	LMT, LEV, VPA, CLB RFM	LMT, LEV	GPFA, GSSW	GPFA, GSSW ▾	Normal	Rt FT ▾	Rt FT ▾	90% decrease
11	M/9	7	Atypical absence	OXC, LEV, TPM	OXC, LEV, TPM	GPFA, GSSW	GPFA, GSSW ▾	Normal	Anterior portion of the Lt ▾	Anterior portion of the Lt ▾	60% decrease
12	M/13	9	Head drop	TPA, LMT, LEV, VPA, CLB	TPA, LMT, LEV, VPA, CLB, CBZ	GPFA, GSSW	MSWD	PVL	Rt PT ▾	Not done	99% decrease
13	F/8	0.5	GT	LEV, VPA, TPA, CLB, RFM	LEV, VPA TPA, CLB, RFM	GPFA, GSSW	Lt LPFA	FCD in Rt cingulate gyrus	Rt T ▾	Rt T ▾	90% decrease
14	F/4	0.2	GT, SMA seizure	ZNS, CLB, RFM	ZNS, CLB, RFM	GPFA, GSSW	GPFA, GSSW ▾	Mild brain atrophy	Rt PQ ▾	Rt PQ ▾	No change

*F, female; M, male; Lt, left; Rt, right; GT, generalized tonic; GTC, generalized tonic clonic; CBZ, carbamazepine; CLB, clobazam; LEV, levetiracetam; LMT, lamotrigine; OXC, oxcarbazepine; RFM, rufinamide; TPM, topiramate; VGB, vigabatrin; VPA, valproate; ZNS, zonisamide; GPFA, generalized paroxysmal fast activity; GSSW, generalized slow sharp and wave; LPFA, localized paroxysmal fast activity; MSWD, multifocal sharp and wave discharge; SWD, sharp and wave discharges; ▾, reduction in frequency; F, frontal; P, parietal; O, occipital; T, temporal; H. hemisphere; PQ, posterior quadrant; FCD, focal cortical dysplasia; PVL, periventricular leukomalacia; WMH, white matter hyperintensity; ▾, hypometabolism in PET and hypoperfusion in SPECT; ▴, hyperperfusion*.

### EEG Acquisition and Preprocessing

Surface-EEG data were recorded from patients in their preoperative and around 3-month (average 86.4 days) postoperative states using a 19-channel digital EEG acquisition system (Telefactor, Grass Technologies) at a sampling frequency of 200 Hz. The EEG reference channel was set as the mean recording value of all 19 channels, as this approach has been suggested as a practical compromise for reducing the confounding effect of brain activity (24). Then, the data were bandpass filtered (third order Butterworth, zero-phase shift digital filtering) on frequency bands of 1–70 Hz and notch filtered at 60 Hz to remove line noise during export process from the EEG system.

Because the subjects involved in this study were all having refractory generalized epilepsy, it was challenging to select a long-term contiguous interictal EEG recording without dysfunctional signal effects. However, a short epoch, 1-s interval, could easily avoid artifacts and abnormal effects, while also balancing signal stationarity. Generally, the brain network modeled by correlation matrices from EEG data shows variability with sampling epochs shorter than 20 s, which rapidly stabilizes with increasing epoch length. The stable network template usually emerges after 100 s, and a more precise network pattern will form with the longest epoch length possible (25–27). Hence, we randomly selected 360 non-epileptic EEG epochs with intervals of 1-s on preoperative and 3-month postoperative recording on patients’ wakeful and rest state to ensure the stabilization of raw data as well as non-artifact effects. The sampling data were then filtered into five separate frequency bands: delta (1–4 Hz), theta (4–8 Hz), alpha (8–13 Hz), beta (13–32 Hz), and gamma (32–55 Hz), to determine each band’s association with distinct network and cognitive functions (28). The filtering tool employed was a finite-impulse response, phase-invariant digital filter of EEGLAB (version 13.6.5b), which was running under MATLAB R2015b. Depending on the filter bandwidths, the filtering order was set from 85 to 661, which could achieve strong suppression of the out-of-band signals with sharp dropoff.

### Network Construction and Visualization

**Figure 1:**
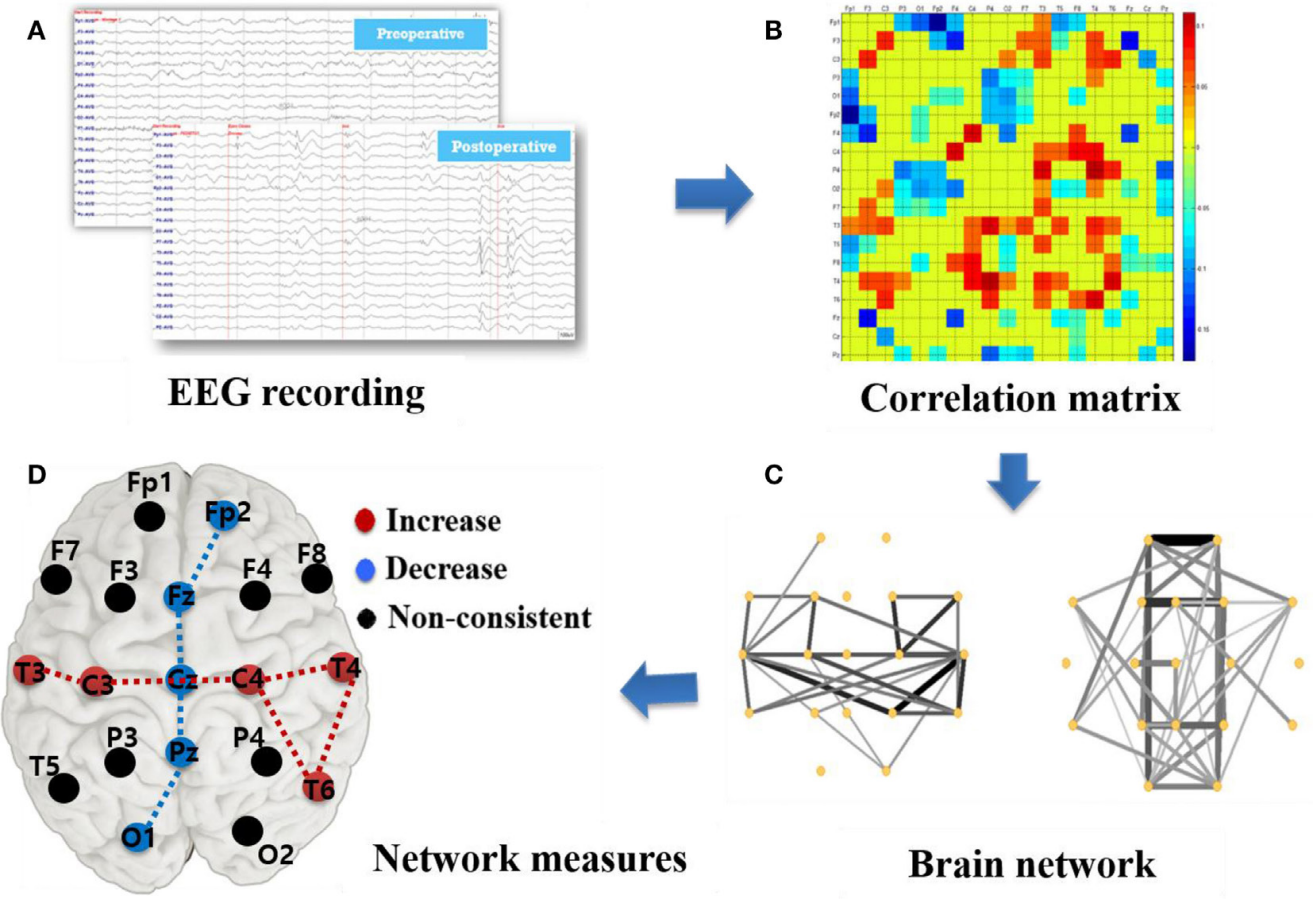
Illustration of network construction and analysis based on surface electroencephalographic (EEG) recording. **(A)** EEG recording: 19 channels surface-EEG recording with a sampling frequency of 200 Hz. **(B)** Correlation matrix (MATLAB pcolor plot): correlation coefficients among 19 channels with the diagonal elements set as 0. **(C)** Brain network plots: the edge strength is represented as thickness of the line. **(D)** Graph measures: calculation of brain network parameters, such as degree, betweenness centrality, cluster coefficient, characteristic path length, and efficiency.

Figure 1 describes the basic analysis process in this study beginning with EEG recording and data acquisition as shown in Figure 1A. The Pearson correlation coefficient was calculated using MATLAB *corrcoef* function (see Supplementary Material Formula 1) between two time-series to specify the relevance among EEG signals recorded from 19 channels. Undirected correlation matrices were built by these correlation coefficients, as described in Figure 1B with a color code representing various correlation strengths. The correlation coefficients lacking significant coupling (*p* > 0.05) as well as the diagonal matrix elements were set to 0.

Next, we plotted a visualization of functional connectivity network as in Figure 1C by employing Pajek.^1^ Only the top 30% of high-strength connections in the weighted cross-correlation matrix were plotted to better visualize the CC effects. Besides, upper 20% and 50% connections were respectively plotted in Supplementary Figure S1 based on the pre- and post-operative network for reference. However, this thresholding was done strictly for illustration purpose only and we used the whole weighted connectivity matrix for further graph metric calculation. To assess the alteration caused by CC, we subtracted the preoperative matrix from the postoperative matrix and plotted the results to better visualize a network change.

We calculated several local and global network measures as indicated in Figure 1D. The local measure, betweenness centrality (BC) and degree based on the weighted correlation matrix, were calculated to investigate the local brain status and its relevance with surgery effects. In this study, “nodes” represented the locations of 19 surface electrodes and “edges” indicated the associativity among them. To quantify the importance level of a specific node in the brain network, the measure BC is related to communication processes and is used to quantify the proximity of each node to the rest of the network (14), whereas the degree was computed as the sum of the nodes’ edges. Furthermore, “hubs” calculated from BC and degree is used to evaluate the influence of several primary nodes on the network. The hubs have a central position in the network as they form many connections with other nodes and connect different modules. Hubs are crucial for efficient communication owing to their extensive connectivity and association with increased synchrony in the network. BC calculation was based on weighted matrices, which overcome the problem of a subjective factor by artificially setting a threshold and also provides a more realistic representation of functional networks (17). The calculation was implemented using the codes from the Brain Connectivity Toolbox.^2^ We then plotted the difference between preoperative and postoperative data in boxplots with OriginPro 8 and the brain pattern expression with color scale electrodes.

In addition, global measures, such as characteristic path length (CPL), global/local efficiency (GE/LE), and clustering coefficient, were calculated from the binary matrices by the code published by Nathan Cahill (MathWorks MATLAB Central) to assist in comprehending the CC effects on the global brain network pattern. To avoid spurious weak connections such as noise, which potentially influence network construction (17), the global network measures were computed on binary matrices. Those measures were obtained by thresholding the matrices at the median value. The diagonal elements were always set to 0. Both the weighted and binary matrices were calculated for each 1 s of EEG data selected and then averaged to ensure data stability.

Global network parameters could be classified into segregation and integration for the insights they deliver (29). Segregation refers to the degree to which a network as a whole becomes interconnected and exchanges information. An important measure of segregation is the clustering coefficient, which is used to evaluate the ability of the network to share specialized information. Integration evaluates the capacity of the network as a whole for inner-exchange information. One of the most commonly used integration parameters is path length, which is the sum of the edge amount of communication among more than three nodes. Another method similar to path length is efficiency, which is almost the inverse of integration and is used to assist in determining the representation of a network’s integration. A short CPL, a low clustering coefficient, and a high GE/LE generally represent a small-world topology of the network and characterize an optimal organization for communication efficiency (20).

## Results

### Network Effects Visualization

**Figure 2:**
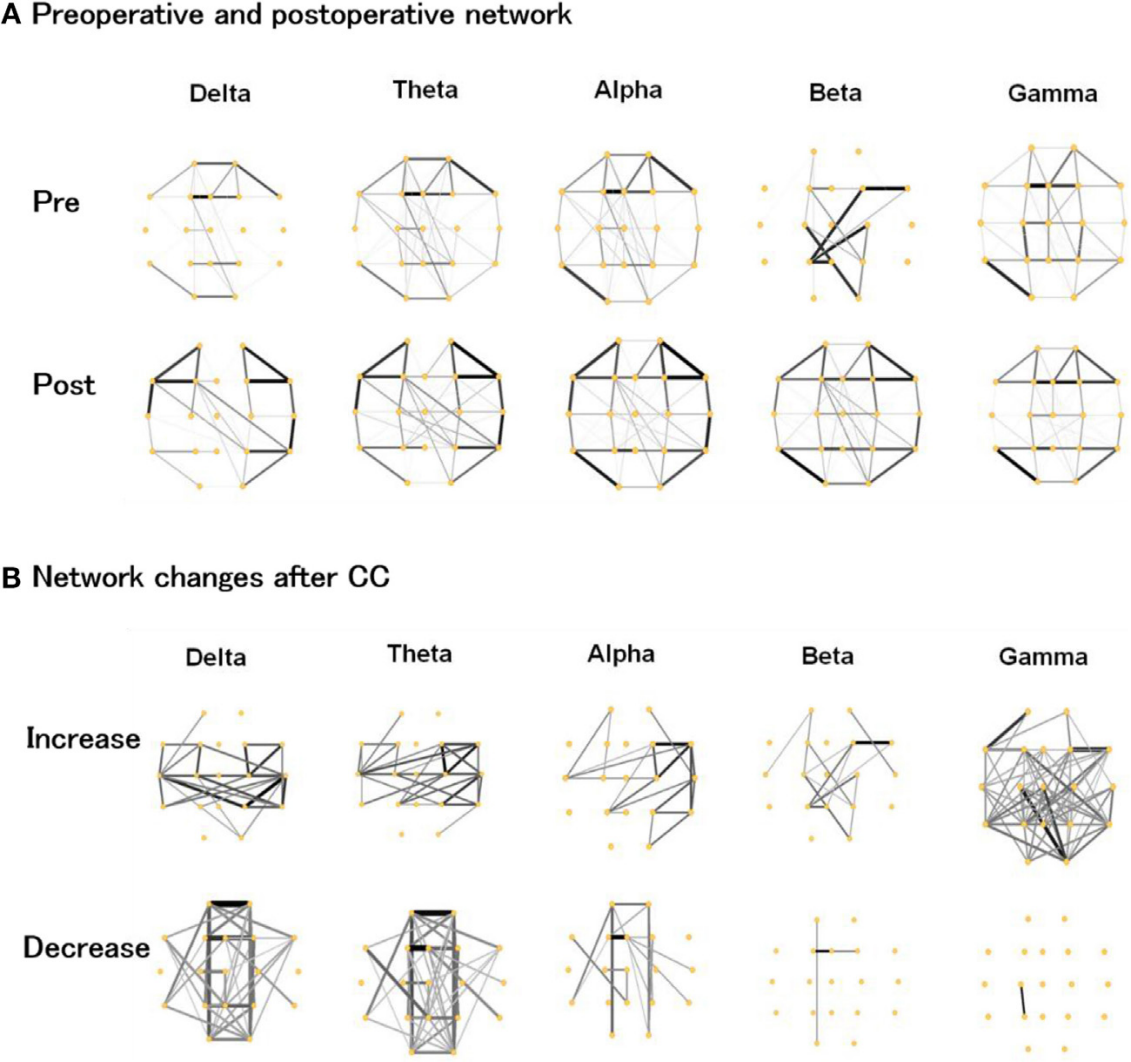
**(A)** Preoperative and postoperative functional networks, **(B)** increased and decreased network connections after corpus callosotomy (CC) in the delta, theta, alpha, beta, and gamma bands. The thickness and gray scale indicates the strength of connections.

As shown in Figure 2A, the preoperative networks showed strong connections around the corpus callosum, although there were slight discrepancies across different frequency bands. After CC, connectivity around paramedian regions was evidently reduced, while the lateral regions expressed an increased connection density. Changes that transformed the brain network into a more homogenous state. Nevertheless, there were still some weak connections spanning the callosum region, maintaining interhemispheric network communication.

We plotted the preoperative and postoperative differences in Figure 2B to better visualize the changes caused by CC. In the delta and theta band, the pattern of increase and decrease was similar and indicated a notable increase in lateral regions with a clear reduction around the paramedian regions. There was a slightly weaker reduction around the paramedian regions in the delta and theta bands. In the alpha band, the right hemisphere showed increased connectivity. In the beta and gamma band, the strength of most connections was enhanced after CC, which was in contrast to the lower frequency bands.

### Local Network Measures Based on Weighted Matrices

#### Postoperative Changes in BC

**Figure 3:**
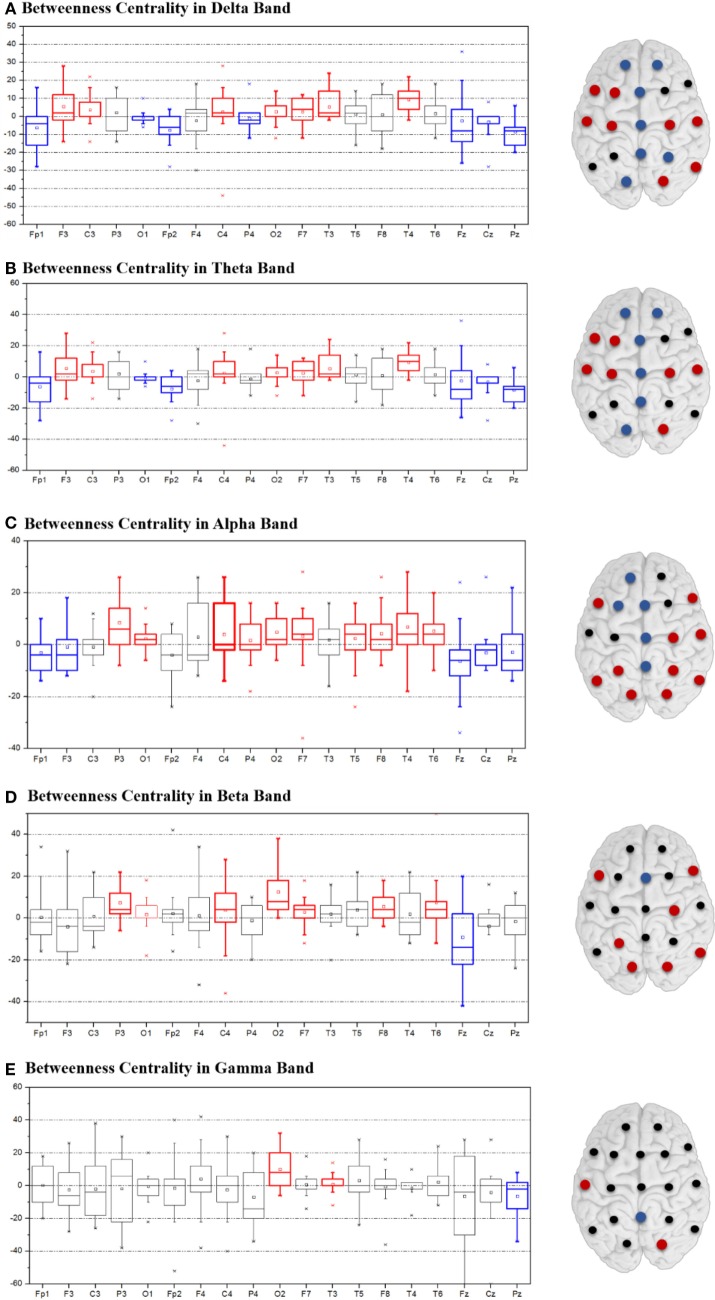
Variation of betweenness centrality between postoperative and preoperative states in the delta **(A)**, theta **(B)**, alpha **(C)**, beta **(D)**, and gamma **(E)** bands. Variation is indicated by colored dots: red denotes increased degree values, blue decreased values, and black non-consistent changes.

Figure 3 depicts the change of BC after CC as calculated by subtracting the postoperative BC values from the preoperative BC, and the change of degree was plotted in Supplementary Figure S2. With respect to the delta, theta, and alpha band, the intensive blue color in Figures 3A–C aligns at the midline regions and corresponds to a significantly decreased BC value. In contrast, the lateral regions of both hemispheres showed an increase in BC. The trend for BC variation in the beta and gamma bands was not statistically significant and was inconsistent between patients (Figures 3D,E). Values for each degree variation are summarized for all the five frequency bands by boxplots (Figure 3).

#### Postoperative Changes in Hub Location

**Figure 4:**
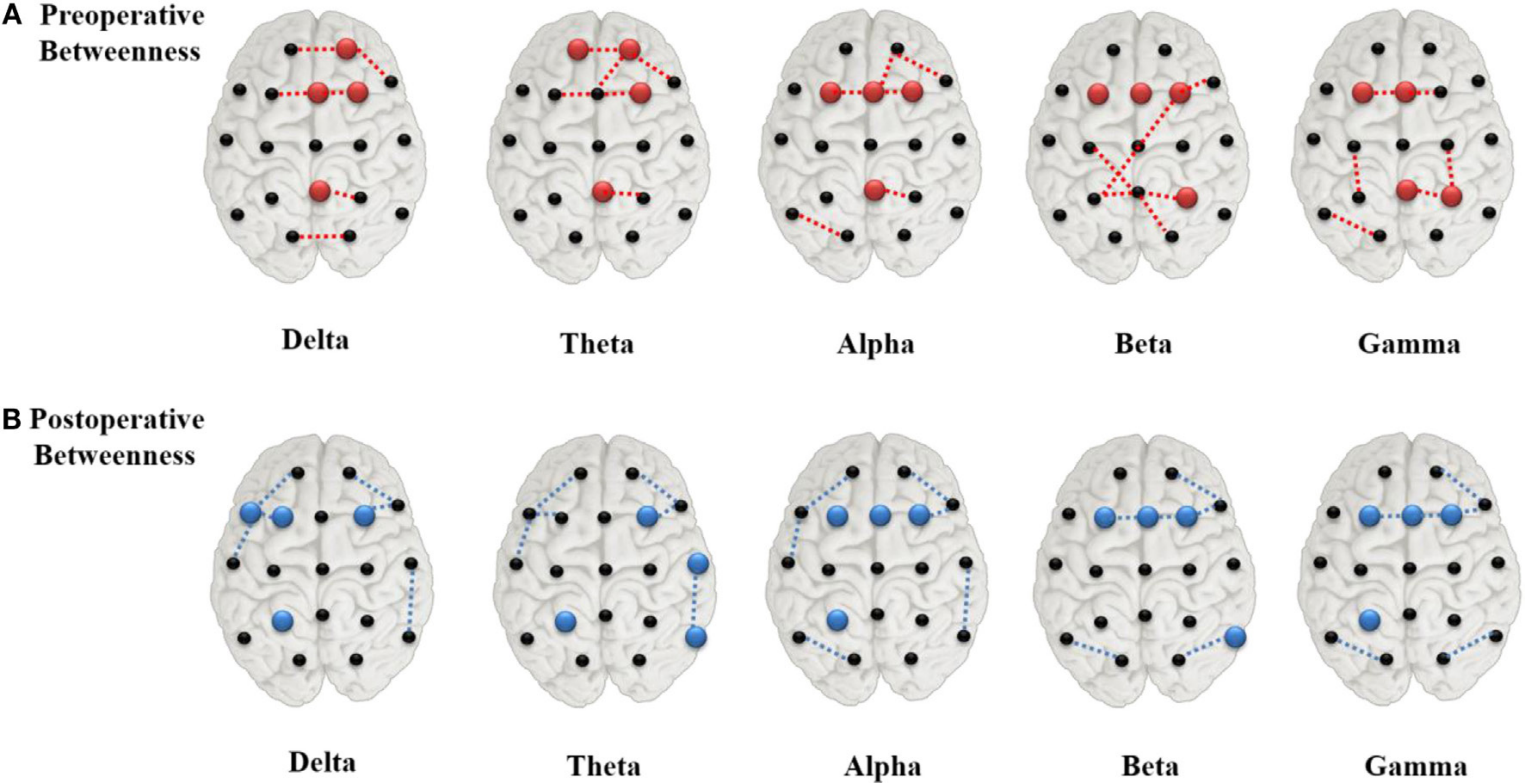
Network hubs derived from betweenness centrality in the preoperative **(A)** and postoperative **(B)** states. The red color dots indicate the preoperative hubs, whereas the blue ones for the postoperative, and the dashed lines represented the top four strongest connections.

The value ranking of the local measures was listed in Supplementary Figure S3. We selected the four channels with the largest BC values as the hubs, which, in preoperative state of the delta and theta band, were Pz, Fp2, Fz, and F4 (Figure 4A). The alpha, beta, and gamma band shared two of these hubs, Fz and F3. T3 and T4, which were at the lateral areas, showed the lowest BC values in almost all frequency bands. Other lateral channels (F7, F8, T5, and T6), also had clearly lower BC values than other channels. After surgery, hubs in the delta and theta band shifted to more lateral regions (Figure 4B), while the hub positions in the other three bands were changed marginally.

### Global Network Measures Based on Binary Matrices

The clustering coefficient showed the trend of decrease in the delta band. However, there was no change in other bands. CPL decreased in the gamma band, but did not change in other bands. Corresponding to the decrease of CPL in the gamma band, GE was slightly increased. There was no significant change in LE or in GE at other frequency bands.

## Discussion

In this study, we aimed to investigate the electroencephalography connectivity in patients with LGS before and after undergoing CC. Since corpus callosum is a major interconnection between the two hemispheres of brain and it facilitates large part of interhemispheric communication, one may expect reorganization of functional connectivity by disconnecting it surgically. First, we visualized the functional networks of patients’ preoperative and postoperative states to examine the effects of CC. Then, local graph measures, including BC, and hubs were calculated on the network at the local level. In addition, several global graph measures, including CPL, global/local efficiency (GE/LE), and clustering coefficient, were also calculated.

### Network Pattern Changes by Hub Disconnection

As nodes with extensive connections and a central position in the network were identified as “hubs,” the removal or alteration of these regions would cause a considerable impact on the network (19, 30). As depicted in Figure 4A, areas of the paramedian regions acted as hubs in the global network during the preoperative state. Severing their interhemispheric connectivity *via* CC eventually led to significant changes in network connectivity, and seizure reduction after surgery may have resulted from disconnection of these hubs by limiting the epileptic activities. After surgery, the hubs near the paramedian regions disappeared in the delta and theta bands (Figure 4B) and new hubs emerged in more lateral regions. Hubs from degree statistics also showed similar varying rule with the BC at pre- and post-operation (see Supplementary Material Figure S4).

The change of hub location directly transformed the global cerebral connective state as shown in Figure 2. All 14 subjects showed consistent and notable brain pattern differences in the comparisons of their preoperative and postoperative network plots. The paramedian area of the brain in the preoperative state showed intensive connectivity while patients were having generalized refractory epilepsy. After a successful CC operation, the connective strength of these regions was evidently weakened and the global network connection transformed to a more uniform state. These phenomena appeared in all EEG frequency bands (1–55 Hz), which could be generally interpreted as the impact of CC on brain pattern variation in patients with LGS being a broadband effect. However, in the postoperative network plot after CC, partial connections spanning the callosum were still observed, indicating basic information exchange between the two hemispheres remained (Figure 2A). According to topographic studies of interhemispheric fibers, the cerebral hemispheres are interconnected mainly *via* three commissures: the corpus callosum, the anterior commissure, and the dorsal hippocampal commissure (6). In addition, other indirect connections, such as through brainstem, may also work for the interhemispheric communication. Our visualization results showed that CC greatly suppressed brain activity across the surgical area due to disconnection of the corpus callosum, but did not completely eliminate interhemispheric network communication.

### Local Network Measure

Local network measure was calculated in this study based on weighted matrices, which was more sensitive and accurate on recording and capturing information in certain frequency bands and revealing local network features in the cerebrum. Because BC is an effective tool for evaluating the level of influence specific nodes have on the network, they were calculated for a quantitative analysis of the network in local regions. We found that the BC values along the paramedian area decreased postoperatively, with corresponding increases in the lateral regions of both hemispheres in the delta, theta, and alpha bands (Figure 3). These results are consistent with the pattern variation in the graphical representation of brain network described earlier. Reduced BC values in the paramedian brain region means that the significance of this region to the network was weakened, whereas the increased values in more lateral region indicated enhanced activity in these areas leading to greater influence on the areas in their vicinity.

### Global Network Measures

Global network measures including clustering coefficient, CPL, and efficiency were computed to assist in observing the overall impact of CC (see Supplementary Material Figure S5). These metrics were calculated from binary matrices to better reject spurious connections. The average clustering coefficient defines the local segregation property of the network, while the CPL and efficiency are measures of global integration. Long paths in the network primarily influence CPL, whereas short paths are primarily represented by GE. After CC, we observed trend of GE increase in the gamma band with CPL decrease in the delta and gamma bands, which indicates optimal integration between small cliques in the global brain network. Taken together with these CPL and GE results, the lower clustering coefficient value observed in the delta band indicated that the brain network converted into a small-world network, which is a more efficient topology (11). It is commonly thought that such an organization reflects an optimal balance of functional integration and segregation (31). Therefore, the postoperative emergence of a small-world network may be connected with improved clinical conditions as well as the recovery process conveyed by CC in patients with LGS. The previous network studies on generalized seizures also found an ictal network with a more regular topography compared to the interictal epoch (32). Global network transfer was found to indicate a less synchronizable network in the interictal period with normalization of the network configuration occurring after ictal termination (33), which corresponds to a decreased clustering coefficient and CPL. As seizure termination approached, the dominant and highly regular subnetwork in the corpus callosum disintegrated into smaller subnetworks within hemispheres, forming a general network pattern that was more random and more closely approximating a small network structure.

### Summary

Clinical outcomes of all 14 LGS patients improved after CC (Table 1). The preoperative state showed a more regular network configuration with a higher clustering coefficient, CPL, and a lower efficiency in specific frequency ranges. These parameters changed substantially after CC and resulted in a less synchronizable network with more of a small-world topology. This network pattern change demonstrated that CC could efficiently alter brain network topology.

One surgical treatment option for patients with LGS is the resection of the epileptogenic zone, which from a network point of view, is the removal of pathologic hubs that generate ictal discharges (34). However, in patients with refractory LGS along with generalized epileptiform discharges and no focal imaging findings, it is usually challenging to identify the precise location of the epileptogenic zone. CC dissects the interhemispheric fissure, dividing the corpus callosum at its midline. This is equivalent to disconnecting the preoperative hubs that may spread ictal discharges. We found that the strong and dense connections near the midline were decreased and became more homogeneous after CC. We also found that the hubs shifted more laterally, which was responsible for interhemispheric spread of ictal waveforms near the midline. These results verify that CC is a very effective surgical treatment option in patients with LGS showing no focal pathologic regions.

However, there are some limitations to this study. First, although various studies have confirmed that brain activity in the gamma band is epilepsy-related (35), other studies have suggested that surface-EEG recording with frequency higher than 20 Hz are vulnerable to recording myogenic artifact (36) and saccadic spike potentials (37). To avoid these potential confounders, future studies should adopt intracranial EEG recordings, as well as auxiliary techniques (e.g., MRI and SPECT). Second, possible effects from age and sex were not taken into account in this study, which means subtle effects on the data of a non-uniform subject group may have affected the reliability of our analyses. Third, since we did not compare the results with the control group, we cannot assure whether the direction of postoperative changes is associated with the normalization or not. Considering these limitations, the results of this study should be interpreted with some caution.

## Ethics Statement

Patients provided written formed consent and children assented as appropriate for age and level of development. All procedures were approved by the institutional review boards.

## Author Contributions

J-GL and DL contributed equally to the work and designed the study; DL collected the data; J-GL processed the data and wrote the manuscript; DL, SY, HK, and N-YK revised the manuscript.

## Conflict of Interest Statement

The authors declare that the research was conducted in the absence of any commercial or financial relationships that could be construed as a potential conflict of interest.

## Footnotes

^1^http://pajek.imfm.si/doku.php.

^2^http://www.brain-connectivity-toolbox.net/.
