# Supplementary material for: Electroencephalography Network Effects of Corpus Callosotomy in Patients with Lennox–Gastaut Syndrome
Source: Front Neurol. 2017 Sep 4;8:456. doi: 10.3389/fneur.2017.00456 (PMC5591410; doi:10.3389/fneur.2017.00456)
Supplement: Supplementary file 1 [file data_sheet_1.docx]

Supplementary Material

Functional Network Effects of Corpus Callosotomy in Patients with Lennox-Gastaut Syndrome

Jun-Ge Liang^†^, Dongpyo Lee^†^, Song Ee Yoon, Heung Dong Kim*, Nam-Young Kim*

^†^ The authors contributed equally to the work of this manuscript.

*** Correspondence:** Heung Dong Kim: [hdkimmd@yuhs.ac](mailto:hdkimmd@yuhs.ac), Nam-Young Kim: nykim@kw.ac.kr

# Supplementary formulas

Supplementary formula 1. Pearson zero-lag correlation coefficient. For two time-series *A* and *B* with *N* data points each, the Pearson correlation coefficient is ($\text{ρ}$) is computed as:

$$\rho= \frac{1}{N-1}\sum_{i=1}^{N} \left( \frac{\bar{A_{i}-\mu_{A}}}{\sigma_{A}} \right)(\frac{B_{i}-\mu_{B}}{\sigma_{B}})$$

where $\text{μ}_{\text{A}}$ and $\text{σ}_{\text{A}}$ are the mean and standard deviation of *A*, respectively, and $\mu_{B}$ and $\sigma_{B}$ are the mean and standard deviation of *B*.

# Supplementary Figures


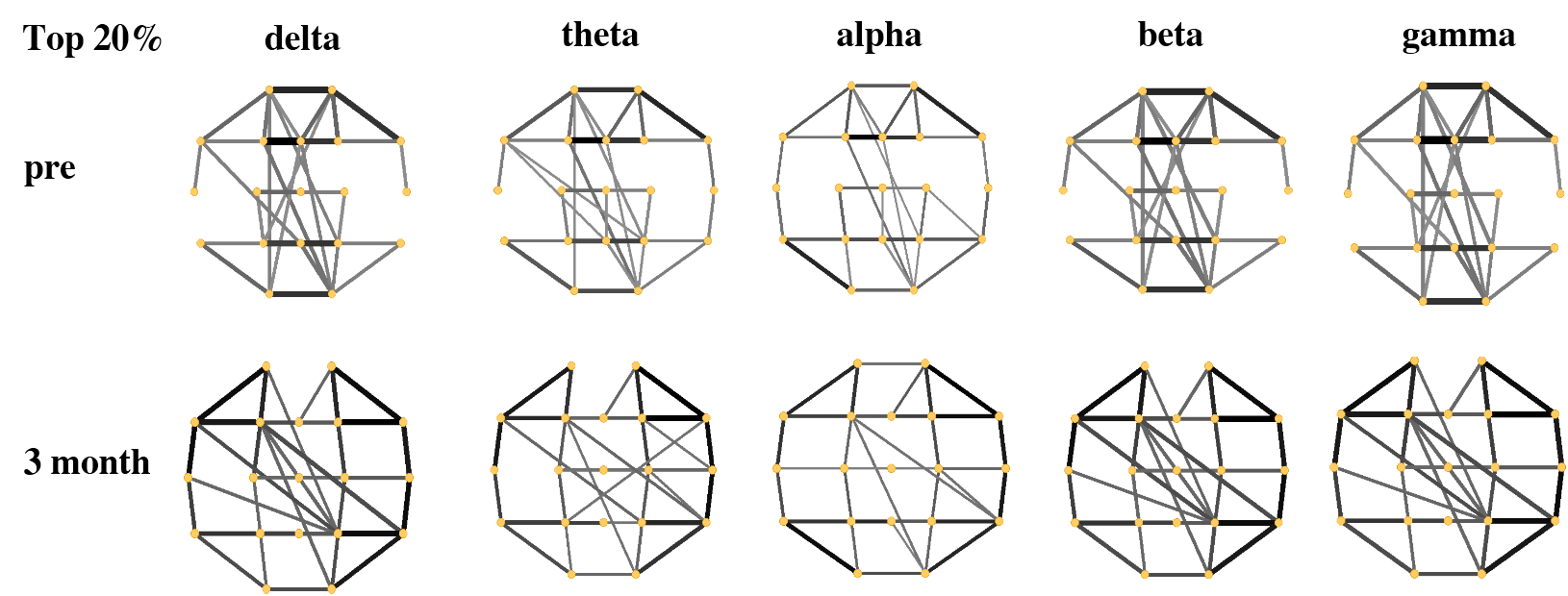

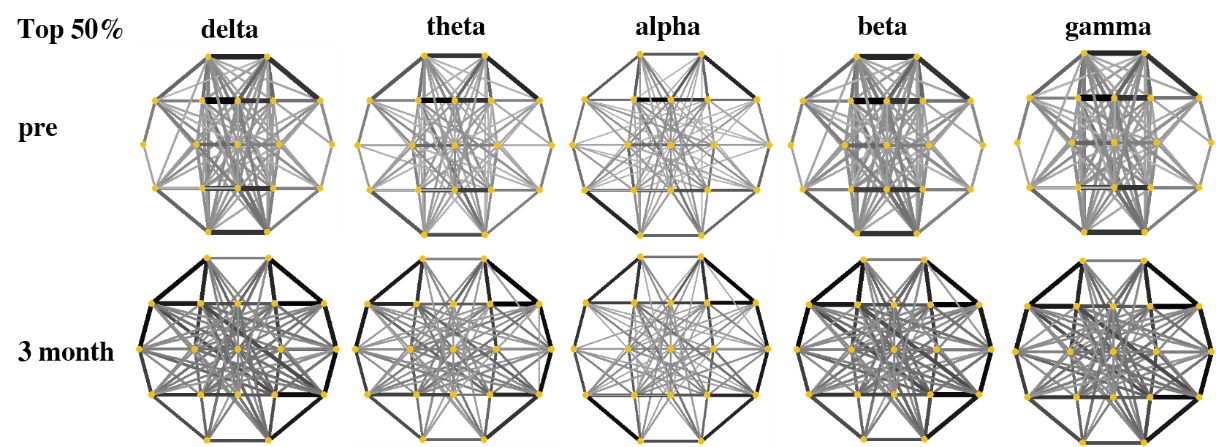


**Supplementary Figure 1.** Preoperative and postoperative functional network plots based on top 20% and 50% threshold.


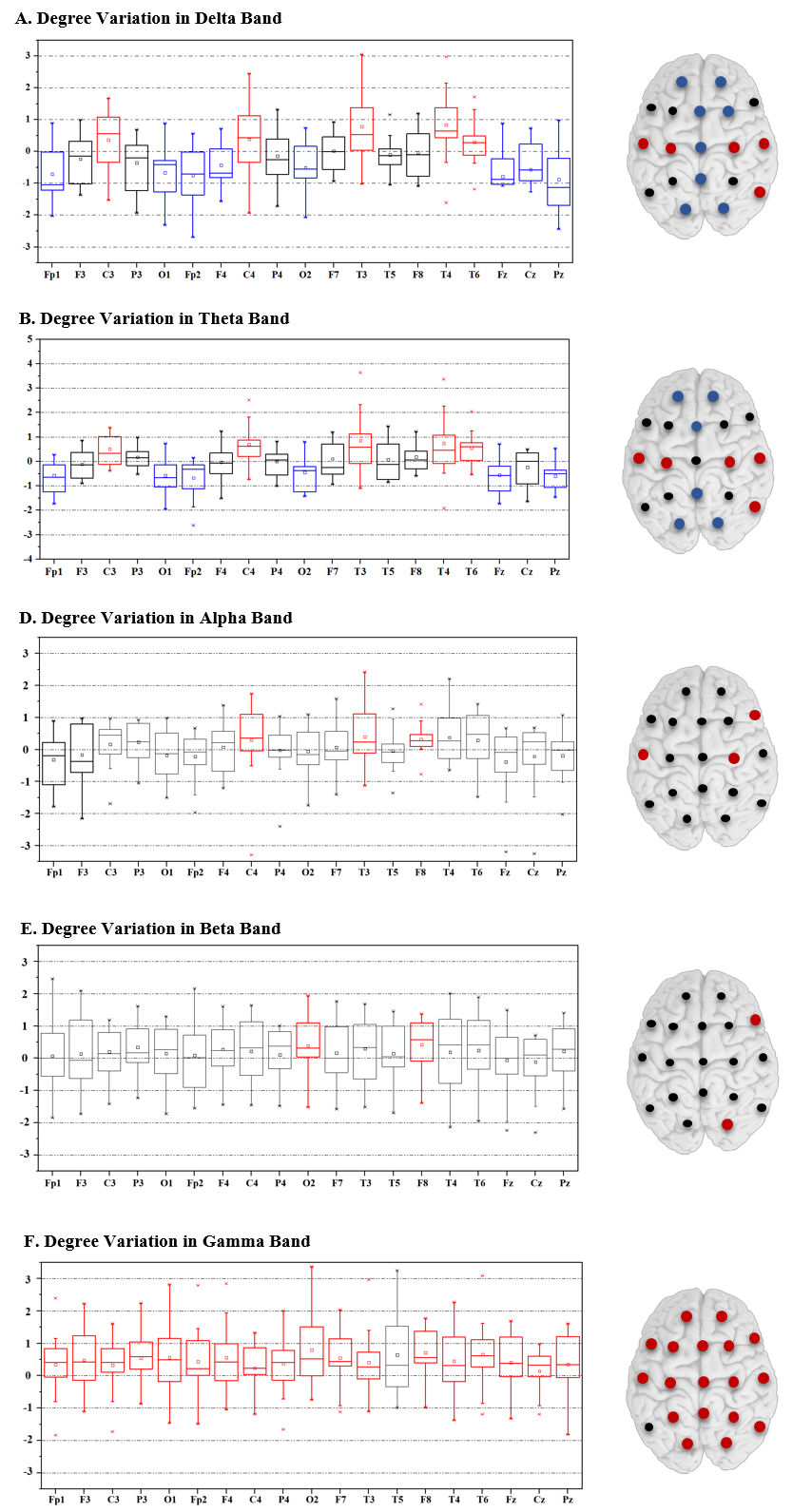


**Supplementary Figure 2.** Degree variation between postoperative and preoperative states in the delta (A), theta (B), alpha (C), beta (D), and gamma (E) bands. Variation is indicated by colored dots: red denotes increased degree values, blue decreased values, and black non-consistent changes.


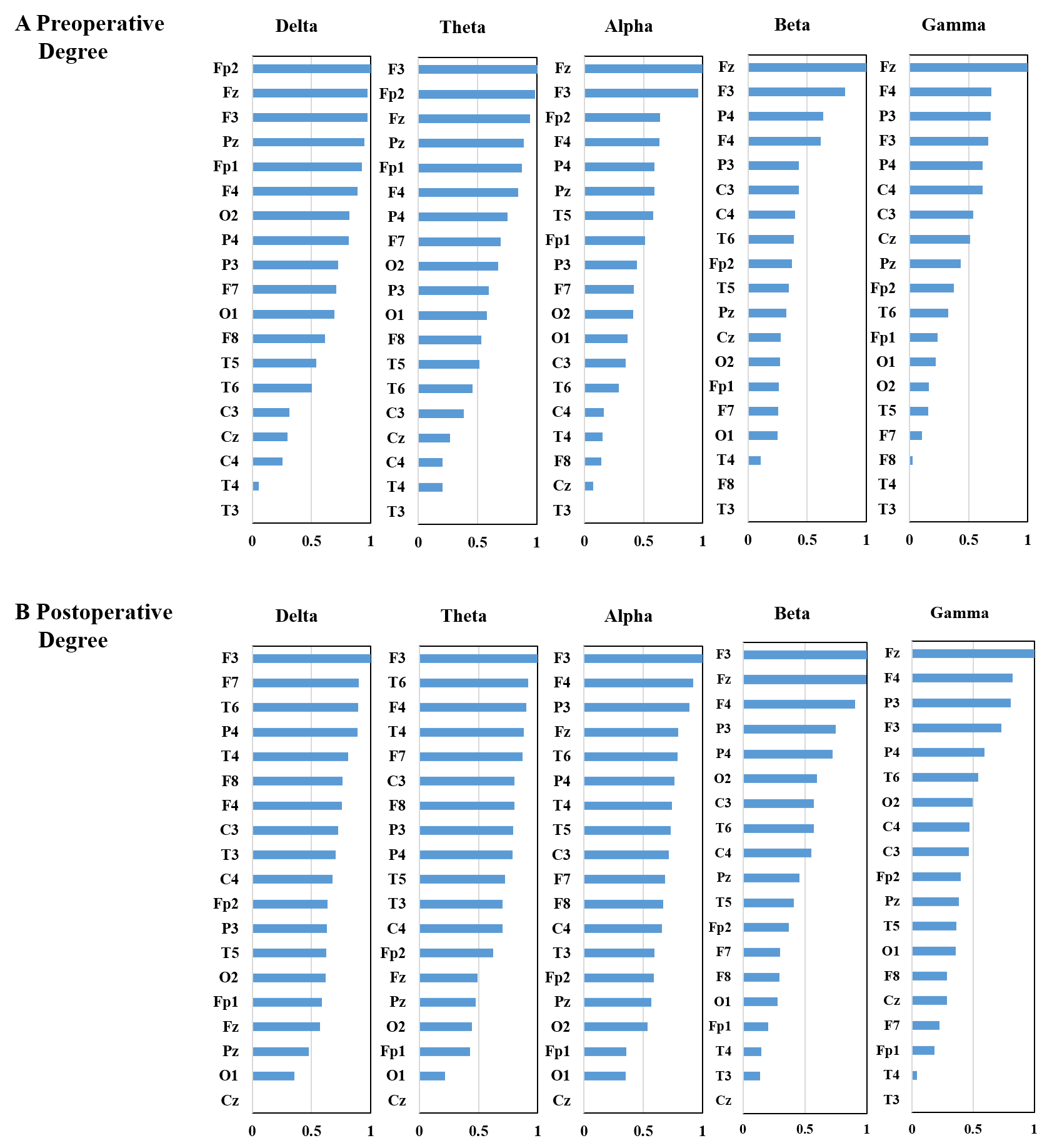


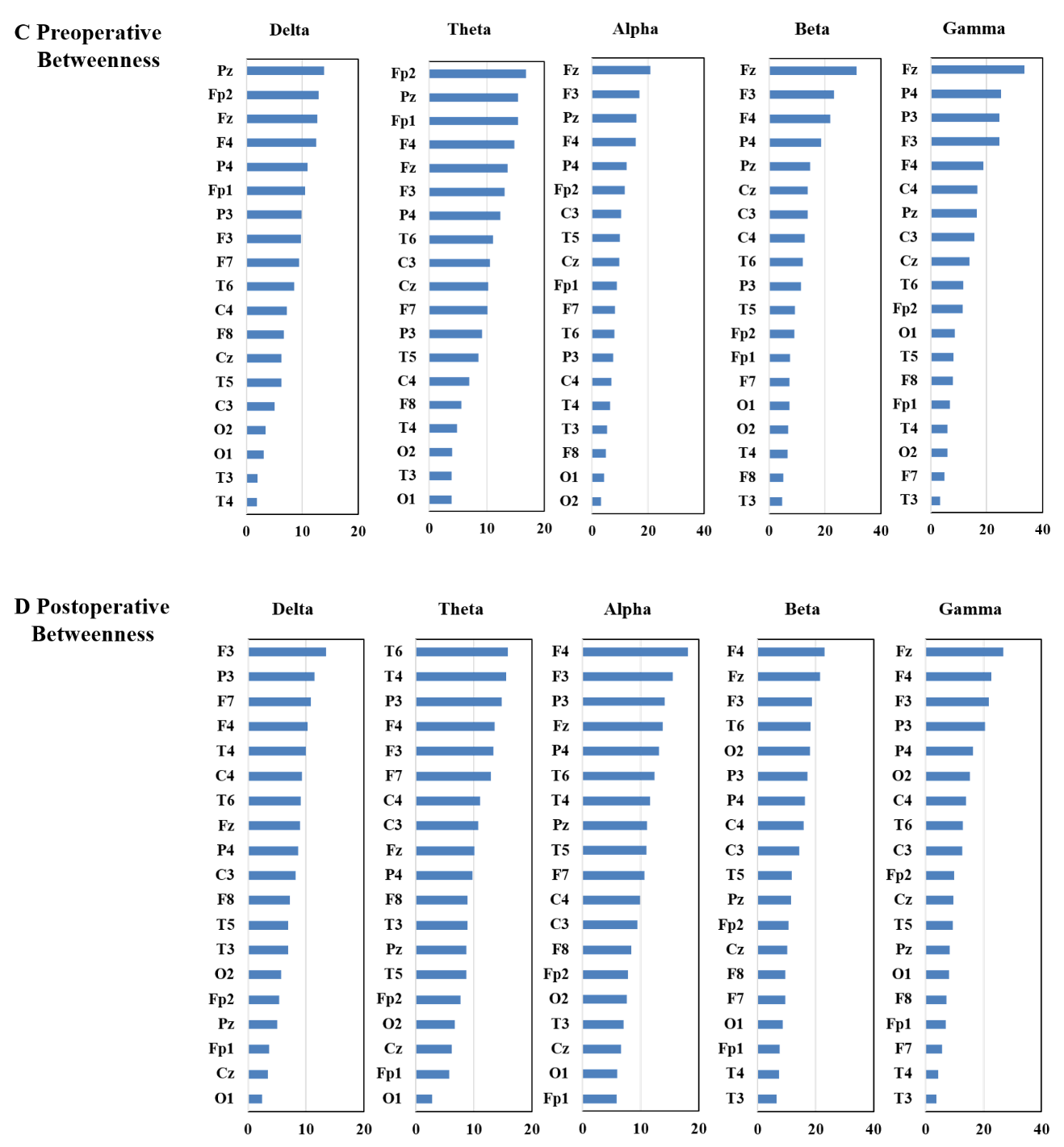


**Supplementary Figure 3.** Ranking of areas for local measures on preoperative and postoperative states in different frequency bands. (A) The normalized degree values ranking on preoperative states and (B) postoperative states. (C) BC values ranking on preoperative states and (D) postoperative states.


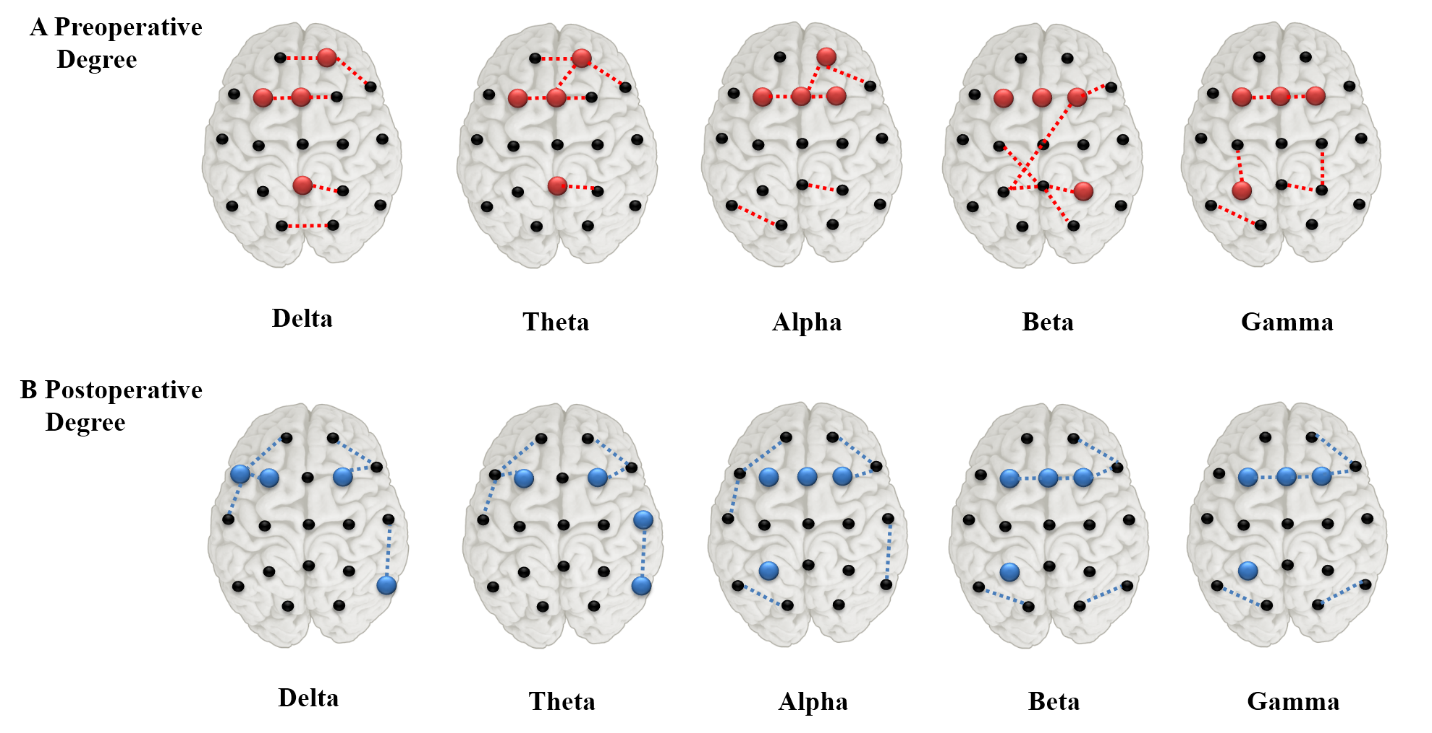


**Supplementary Figure 4.** Network hubs derived from degree in the preoperative (C) and postoperative (D) states. The dashed lines represented the top four strongest connections.


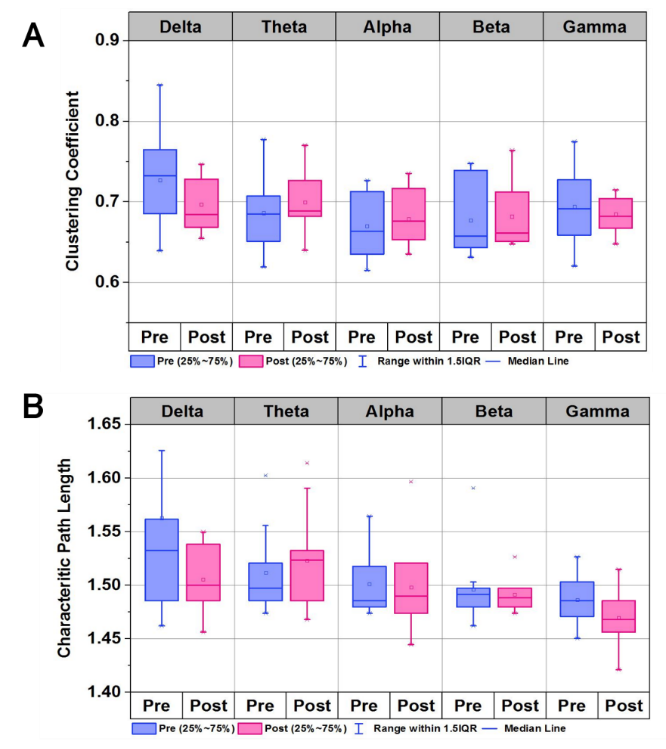

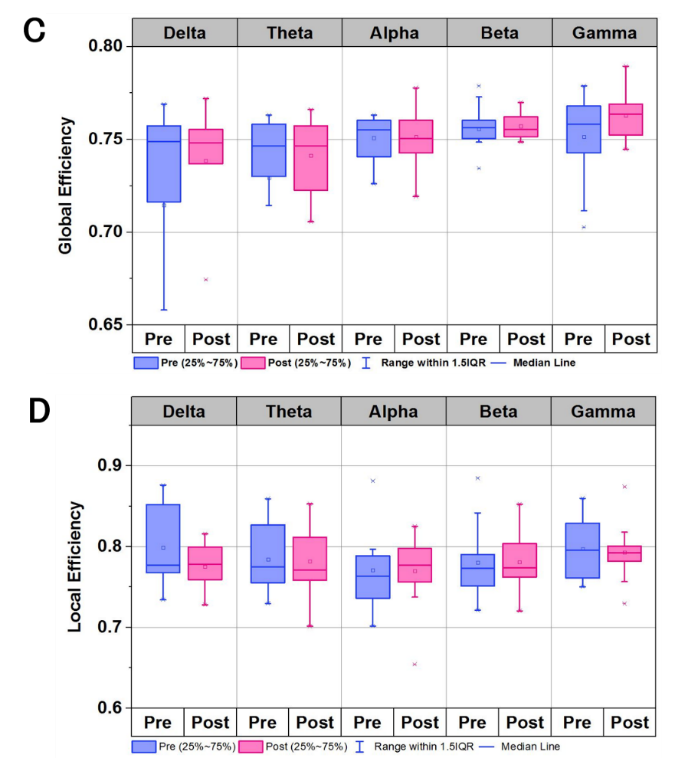


**Supplementary Figure 5.** Global network measure changes based on binary matrices among the fourteen patients. (A) clustering coefficient, (B) characteristic path, (C) global efficiency, and (D) local efficiency.
